# Estimating Hardening Soil-Brick model parameters for sands based on CPTU tests and laboratory experimental evidence

**DOI:** 10.1038/s41598-024-65789-5

**Published:** 2024-07-02

**Authors:** Andrzej Truty

**Affiliations:** Faculty of Civil Engineering, Cracow University of Technology, Warszawska 24, 31-155 Cracow, Poland

**Keywords:** Hardening soil model, Soil-structure interaction, CPTU tests, Calibration procedures, Engineering, Civil engineering

## Abstract

The Hardening Soil-Brick model for soils is designed to carry out complex numerical analyses of soil-structure interaction problems taking into account strong stiffness variation in the range of small strains. However, to calibrate its parameters advanced triaxial and oedometric tests are required. In case of uncemented sands laboratory testing is usually difficult. Therefore, to facilitate calibration procedures, a CPTU based method, enhanced by an experimental evidence derived from advanced triaxial drained and oedometric tests, has been proposed and verified. It is shown in the paper that using exclusively the CPTU test results one can calibrate most important model parameters for sands with accuracy that is sufficient for solving real life problems. The major goal of this paper is to identify correlations between all reference stiffness moduli, then verify them, and finally link with the CPTU based identification procedures. It is shown in the paper that such correlations exist and they exhibit very high coefficients of determination. Moreover, as the seismic version of the CPTU test is not often available in the practice, an enhanced procedure for identification of very small strain shear stiffness modulus has been proposed and then verified, using set of the SCPTU tests conducted in Gdańsk sands (Poland).

## Introduction

The Hardening Soil-Brick (HS-Brick)^1^ is the refined version of the well known Hardening Soil-small (HSs) model^2^, designed to represent general soil behavior. In the HS-Brick model the small strain overlay module of the former model version (HSs)^2^ has been replaced by the Brick concept^3^. The brick procedure removes pathological stiffness overshooting effect identified in the HSs version when dealing with the transient loadings^4^. This model plays nowadays an important role in large scale FEM simulations of complex soil-structure interaction (SSI) problems. The major benefit of this model is such that most of its parameters have a clear interpretation and can easily be estimated based on the laboratory triaxial and oedometric test results. However, laboratory tests yield a limited knowledge on the subsoil and are sensitive to the sampling procedures. Therefore, it is quite natural that field tests, mainly the SCPTU and/or SDMT, supplemented by standard subsoil profiling, are frequently used to characterize construction sites. These tests enable one to identify geotechnical layers and describe spatial variability of certain geotechnical parameters. Another important aspect, mainly of the CPTU test, is such that density of measurements is very high. Therefore CPTU test seems to be an optimal tool which may help to identify density probability functions for strength, stiffness and stress history parameters. In fact instead of randomizing direct model parameters one can randomize dimensionless cone and shaft resistances and then using them derive random model parameters. Such an approach has been proposed in Ref.^5^ but reference stiffness moduli ratios $E_0^{\textrm{ref}}/E_{\textrm{ur}}^{\textrm{ref}}$ and $E_{\textrm{ur}}^{\textrm{ref}}/E_{\textrm{50}}^{\textrm{ref}}$ were assumed there as constant values. Results of this study clearly show that these ratios are not constant and correlations exhibiting high values of coefficient of determination can be identified when moduli ratios $G_0^{\textrm{ref}}/E_{\textrm{50}}^{\textrm{ref}}$ and $E_{\textrm{ur}}^{\textrm{ref}}/E_{\textrm{50}}^{\textrm{ref}}$ are related to dimensionless reference secant stiffness modulus $E_{\textrm{50}}^{\textrm{ref}}/p_a$ ( $p_a$ is the reference stress value usually assumed as 100 kPa). To prove that, the approximation formulas for stiffness moduli $G_0$, $E_{50}$ and $E_{\textrm{oed}}$, derived by Wichtmann et al.^6^ from high quality triaxial and oedometric tests, conducted on 19 sands, have been reused to identify these correlations. They were further verified using results of drained triaxial tests conducted on Łódź sands (Poland). In order to link the evidence derived from laboratory tests with the CPTU based identification procedures an enhanced correlation formula has been derived for the very small strain shear modulus $G_0$, following the idea proposed by Ahmed^7^. This formula was verified then using SCPTU test results conducted in Gdańsk sands (Poland).

The article is organized as follows. In “Summary of the HS-Brick model formulation and its parameters” a summary of the Hardening Soil-Brick model and its parameters is given. Then in “Relations between reference stiffness moduli derived from drained triaxial tests” certain correlation formulas for the stiffness exponent *m*, in the range of small and moderate strains, are analyzed. In further subsections correlation formulas $G_0^{\textrm{ref}}/E_{\textrm{50}}^{\textrm{ref}}(E_{\textrm{50}}^{\textrm{ref}}/p_a)$, $E_{\textrm{ur}}^{\textrm{ref}}/E_{\textrm{50}}^{\textrm{ref}}(E_{\textrm{50}}^{\textrm{ref}}/p_a)$ and $E_{\textrm{oed}}^{\textrm{ref}}/E_{\textrm{50}}^{\textrm{ref}}(E_{\textrm{50}}^{\textrm{ref}}/p_a)$ are derived from Wichtmann’s et al. study^6,8^ and then verified for Łódź sands. In addition an effect of fixed constant value of the *m* exponent, which is the HS-Brick model limitation, is studied in a qualitative and quantitative manner. In “Estimating elastic stiffness in the range of very small strains using CPTU tests” and “Stiffness degradation in the range of small strains”, respectively, enhanced correlation formula for the $G_0$ modulus and formula for the $\gamma _{0.7}$ model parameters are derived to be used in conjunction with the standard CPTU test. In “Estimating peak friction and dilatancy angles from CPTU tests” a short summary of the CPTU correlations used to assess peak friction and dilatancy angles for sands is given. Section “Conclusions” summarizes results of this study.

## Summary of the HS-Brick model formulation and its parameters

The complete theory of this model can be found in Ref.^1^, therefore only a brief summary is given in this section.The HS-Brick belongs to the class of isotropic multisurface elasto-plastic models with strain hardening. The two uncoupled plastic mechanisms ie. the shear and the volumetric one (cap) are introduced to reproduce triaxial and oedometric tests with a relatively high level of accuracy. The ultimate limit states are controlled by the standard Coulomb-Mohr and Rankine strength criteria. Elastic behavior is modeled using classical hypo-elastic formulation in which current $E_0$ and $E_{{ur}}$ stiffness moduli are stress dependent, while the stiffness degradation, from $E_0$ to the $E_{{ur}}$ value, is managed using Brick procedure. The whole set of HS-Brick/HSs model parameters can be classified in three major groups characterizing strength and dilatancy, stiffness, and stress history. Strength parameters include the classical peak effective friction angle $\phi ^{\prime }$ and effective cohesion $c^{\prime }$. Dilatancy is described by the dilatancy angle $\psi$. An extra cut-off condition expressed by means of the $e_{\textrm{max}}$ value can be added to cancel excessive volume changes during shearing. Stiffness is characterized by the reference stiffness modulus in the range of very small strains $E_{\textrm{0}}^{\textrm{ref}}$, secant unloading-reloading modulus $E_{\textrm{ur}}^{\textrm{ref}}$, secant modulus $E_{\textrm{50}}^{\textrm{ref}}$, Poisson’s ratio $\nu _{\textrm{ur}}$, stiffness exponent *m*, an equivalent shear strain $\gamma _{0.7}$, used to parametrize secant stiffness $E_{\textrm{sec}}/E_0$ degradation curve, and oedometric tangent stiffness modulus $E_{\textrm{oed}}$ defined at a given vertical stress value $\sigma _{\textrm{oed}}$. Stress history is traced using the assumed overconsolidation ratio (OCR) or the preoverburden pressure (POP) which yields, variable with depth, OCR(*z*) profile.

In this study the $p^{\prime }$ form of the stiffness stress dependency function (the $\sigma _3^{\prime }$ type is available as well in the HS-Brick), compatible with the Wichtmann’s et al. study^6^, is chosen. Therefore current stiffness moduli $E_{\textrm{0}}$, $E_{\textrm{ur}}$, $E_{\textrm{50}}$ are defined as follows

1 $$\begin{aligned} E_0= & {} E_{\textrm{0}}^{\textrm{ref}}\left( \dfrac{p}{\sigma _{\textrm{ref}}} \right) ^m \end{aligned}$$

2 $$\begin{aligned} E_{\textrm{ur}}= & {} E_{\textrm{ur}}^{\textrm{ref}}\left( \dfrac{p}{\sigma _{\textrm{ref}}} \right) ^m \end{aligned}$$

3 $$\begin{aligned} E_{\textrm{50}}= & {} E_{\textrm{50}}^{\textrm{ref}}\left( \dfrac{p}{\sigma _{\textrm{ref}}} \right) ^m \end{aligned}$$

The stiffness exponent *m* is assumed as a constant parameter although it is known that its value is a function of the equivalent shear strain $\gamma$^9^. In case of sands its value varies from $m\approx 0.4$, in the regime of very small strains, up to $m\approx 1$, in the domain of medium and large strains. In order to reduce model complexity this effect is not taken into account but detailed discussion concerning this problem is given in “Estimating stiffness exponent”.

It is worth to mention that in the classical Hardening Soil-small model stiffness moduli vary with the minor effective stress $\sigma _3^{\prime }$. Mapping reference stiffness moduli derived for the $p^{\prime }$ version, to the classical form, can be done assuming stiffness equivalence corresponding to the in situ stress conditions. This yields the following mapping rule

4 $$\begin{aligned} E^{\text {ref},\sigma _3}=E^{\text {ref},p}\left( \dfrac{1+2\;K_0}{3\;K_0} \right) ^m \end{aligned}$$

## Relations between reference stiffness moduli derived from drained triaxial tests

In most practical SSI problems, analyzed using the HS-Brick model, crucial role are playing the three stiffness moduli ie. very small strain stiffness modulus $E_{\text {0}}$, the secant unloading-reloading modulus $E_{\text {ur}}$ and secant modulus $E_{\text {50}}$. Therefore it is beneficial if one could identify relations between their reference values with possibly high level of accuracy. In this section it will be shown that such relations exist but prior their derivation correlations between the stiffness exponent *m*, in the range of very small strains, and exponents ratio $m/\tilde{m}$ ($\tilde{m}$ corresponds to the medium strains range) have to be identified first.

### Estimating stiffness exponent

In paper by Wichtmann et al.^10^ the following correlation formula between the *m* exponent, uniformity coefficient $C_u$ and fines content $\textrm{FC}$ (here $\textrm{FC}$ has to be expressed in decimal notation) was proposed

5 $$\begin{aligned} m=0.4\;C_u^{0.18}\left( 1+0.116\;\ln \left( 1+100\;\textrm{FC} \right) \right) \end{aligned}$$

**Figure 1:**
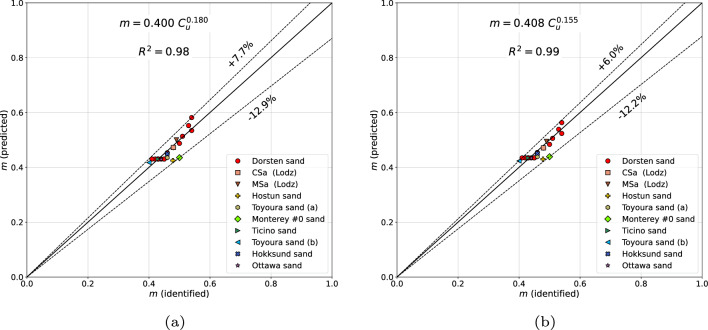
Predicted vs identified stiffness exponents *m* for clean sands using formula (5) (**a**) and formula (6) (**b**).

**Figure 2:**
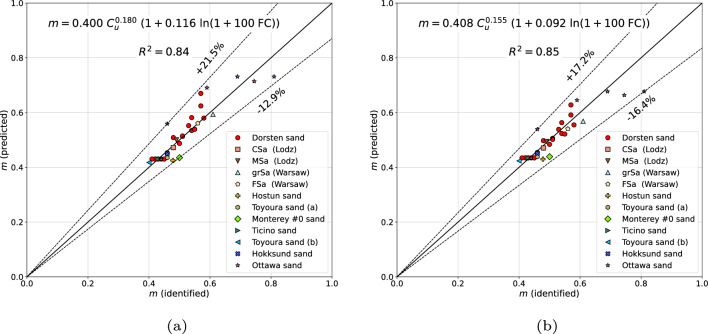
Predicted vs identified stiffness exponents *m* for all sands using formula (5) (**a**) and formula (6) (**b**).

In order to verify this formula on a wide set of the experimental data, sands from several sites, all over the world, have been analyzed. The considered set of tested sands includes Dorsten sand^6^, coarse and medium Łódź (Poland) sands, Hostun sand^11^, Toyoura sand(a)^12^, Toyoura sand(b)^13–15^, Monterey #0 sand^15–17^, Ticino sand^13–15^, Hokksund sand^13–15^ and Ottawa sand^15^. It has to be mentioned that the Monterey #0 and Hostun sands were not active in the optimization procedure but they were considered to identify error margins. As it is shown in Fig. 1a the estimation error, for clean sands, using formula (5), is ranging from $-12.9$ to 7.7%. A slightly better prediction can be obtained with the following formula

6 $$\begin{aligned} m=0.408\;C_u^{0.155}\left( 1+0.092\;\ln \left( 1+100\;\textrm{FC} \right) \right) \end{aligned}$$

for which the estimation error is ranging from $-12.2$ to 6.0% (cf. Fig. 1b). Both formulas (5) and (6) yield a larger scatter for sands with nonzero fines content $\textrm{FC}$ (cf. Fig. 2a,b). However, one may observe that differences between the two formulas are not significant, hence, high accuracy of Wichtmann’s formula (5) is confirmed.

It is well known from the literature^9^ that the power exponent *m* is in fact a monotonically increasing function of the equivalent shear strain. However, in the HS-Brick model, neither in the classical HS-small, this dependency cannot be taken into account and a constant *m* value has to be adopted. Therefore, it is not obvious whether the *m*, defined in the range of very small strains or $\tilde{m}$, defined in the range of medium strains, a fixed exponent $m=0.5$, or an averaged $1/2(m+\tilde{m})$ can be adopted in practice. This problem will carefully be analyzed in “[Sec Sec5]”.

The relation between the $\tilde{m}$, $C_u$ and $\textrm{FC}$, identified on a smaller set of the experimental data, can be approximated by the formula

7 $$\begin{aligned} m/\tilde{m}= 0.374 + 0.087\;C_u \le 1 \end{aligned}$$

**Figure 3:**
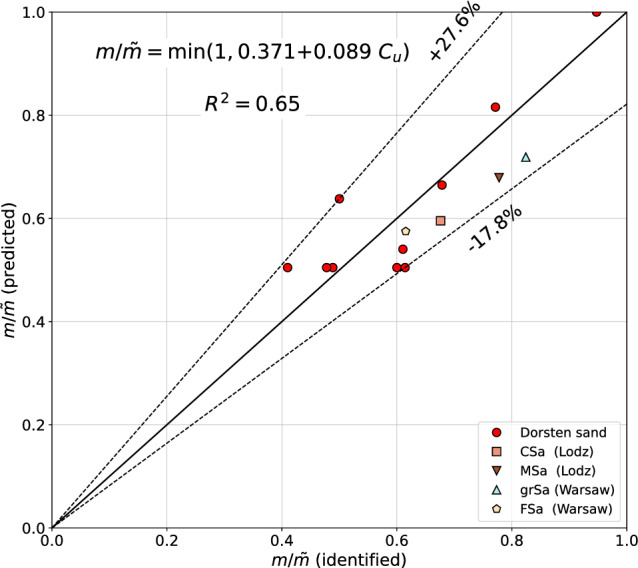
Predicted vs identified $m/\tilde{m}$ ratios (all sands).

Comparizon of the identified and predicted $m/\tilde{m}$ values is shown in Fig. 3. As it was expected the error margins are larger than for the *m* parameter prediction, however, a certain correlation between $m/\tilde{m}$ and $C_u$ exists.

### Relation between reference moduli $G_0^{\textrm{ref}}$ and $E_{50}^{\textrm{ref}}$

The relation between reference stiffness moduli $G_0^{\textrm{ref}}$ and $E_{\textrm{50}}^{\textrm{ref}}$, for a wide range of sandy soils, has been established using results of high quality triaxial tests published by Wichtmann et al.^6^. In the article authors present a complete set of the data in form of the best fit formulas identified for the small strain stiffness modulus $G_0$, secant modulus $E_{\textrm{50}}$ and the tangent oedometric modulus $E_{\textrm{oed}}$, in the wide range of relative densities $D_r$ and initial consolidation stresses $p_{0}^{\prime }$. In the set of 19 tested sands and gravelly sands samples L1..L8 are characterized by the uniformity coefficient $C_u=1.5$ and variable $d_{\textrm{50}}$ diameter ranging from 0.1 to 6.0 mm, samples L10..L16 are characterized by a constant $d_{\textrm{50}}=0.6$ mm and variable $C_u$ ranging from $C_u=2..8$ while samples F2,F4..F6 are characterized by $d_{\textrm{50}}=0.092..0.082$ mm, $C_u=1.5..3.3$ and fine content ranging from 4.4 to 19.6%.

A preliminary study indicated that classifying all tested samples to three independent classes i.e., medium/coarse sands, gravelly sands and fine sands is needed in some cases. More general relation between $G_0^{\textrm{ref}}$ and $E_{\textrm{50}}^{\textrm{ref}}$ for gravelly sands, coarse sands and medium sands, treated as one general group, was also derived. The remaining samples L6..L8 and L13..L16 were classified as gravelly sands, samples L2..L5 and L10..L12 were classified as coarse and medium sands while samples L1 and F2, F3..F6 as fine sands.

It has been found that a strong correlation exists between dimensionless quantities $G_0^{\textrm{ref}}/E_{\textrm{50}}^{\textrm{ref}}$ and $E_{\textrm{50}}^{\textrm{ref}}/p_a$ for all three selected sand classes. All distinct points in the presented $G_0^{\textrm{ref}}/E_{\textrm{50}}^{\textrm{ref}}-E_{\textrm{50}}^{\textrm{ref}}/p_a$ diagrams, were computed using approximation formulas for the $G_0$ and $E_{50}$ moduli, and corresponding stiffness exponents *m* (in Ref.^6^ denoted as $n_{Gd}$) and $\tilde{m}$ (in Ref.^6^ denoted as $n_{Es}$).

In the context of the HS-Brick model formulation the following constraint is mandatory

8 $$\begin{aligned} \dfrac{E_{0}^{\textrm{ref}}}{E_{\textrm{50}}^{\textrm{ref}}}=\underbrace{\dfrac{E_{0}^{\textrm{ref}}}{E_{\mathrm {\textrm{ur}}}^{\textrm{ref}}}}_{> 1} \underbrace{\dfrac{E_{\textrm{ur}}^{\textrm{ref}}}{E_{\textrm{50}}^{\textrm{ref}}}}_{> 2} > 2 \end{aligned}$$

Therefore, the lower bound estimate, for the ratio ${G_{0}^{\textrm{ref}}}/{E_{\textrm{50}}^{\textrm{ref}}}$, takes then the following form

9 $$\begin{aligned} \dfrac{G_{0}^{\textrm{ref}}}{E_{\textrm{50}}^{\textrm{ref}}} > \dfrac{1}{1+\nu } \end{aligned}$$

In order to preserve a certain difference between ${E_{0}^{\textrm{ref}}}$ and ${E_{\textrm{ur}}^{\textrm{ref}}}$ moduli for large $E_{\textrm{50}}^{\textrm{ref}}/p_a$ values, condition (Eq. 9) can be formulated in a more restrictive form

10 $$\begin{aligned} \dfrac{G_{0}^{\textrm{ref}}}{E_{\textrm{50}}^{\textrm{ref}}} > 1 \end{aligned}$$

In general, relation between dimensionless quantities $G_0^{\textrm{ref}}/E_{\textrm{50}}^{\textrm{ref}}$ and $E_{\textrm{50}}^{\textrm{ref}}/p_a$ can be approximated using the following power law

11 $$\begin{aligned} \dfrac{G_0^{\textrm{ref}}}{E_{\textrm{50}}^{\textrm{ref}}}=1+A\;\left( E_{\textrm{50}}^{\textrm{ref}} / p_a \right) ^n \end{aligned}$$

in which coefficients *A* and *n* have to be optimized for each distinct class of sands. Three sets of coefficients *A* and *n*, for gravelly (grSa), coarse/medium (CSa/MSa), gravelly/coarse/medium sands (grSa/CSa/MSa) and fine sands (FSa), are given in Table 1. The first set corresponds to the assumption that modulus $G_{\textrm{0}}^{\textrm{ref}}$ is identified using stiffness exponent *m*, while the $E_{\textrm{50}}^{\textrm{ref}}$, using stiffness exponent $\tilde{m}$, respectively. This assumption can be treated as a consistent one. In the second set reference stiffness moduli are computed using unique, for both of them, stiffness exponent *m*, while in the third set, an arbitrary value $m=0.5$ is assumed. The last two assumptions are verified here as in the HS-Brick model the stiffness exponent *m* is a constant. Coefficients of determination $R^2$ are not given in the table but one can find them in the following figures.

**Table 1:** Optimized values of coefficients *A* and *n* for all classes of sands.

Sand class	A	n	A	n	A	n
grSa	374.3	$-0.826$	567.8	$-0.914$	628.8	$-0.934$
CSa/MSa	560.7	$-0.908$	917.7	$-1.015$	1009.5	$-1.035$
grSa/CSa/MSa	513.2	$-0.890$	845.7	$-0.996$	935.5	$-1.017$
FSa	424.3	$-1.063$	721.2	$-1.190$	464.5	$-1.089$

**Figure 4:**
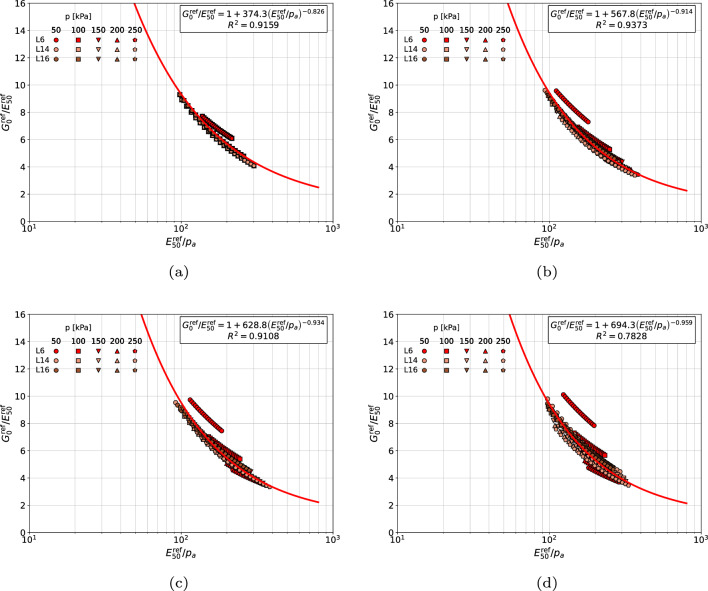
Relations ${G_0^{\textrm{ref}}}/{E_{50}^{\textrm{ref}}}$ vs ${E_{50}^{\textrm{ref}}}/{p_a}$ for gravelly sands (in (**a**) distinct *m* and $\tilde{m}$ values are used, in figure (**b**) $\tilde{m}=m$ is assumed, in figure (**c**) fixed $m=\tilde{m}=0.5$ is assumed, in figure (**d**) the averaged value $0.5(m+\tilde{m})$ is used).

For all considered sands classes, four assumptions, concerning choice of the power exponents *m* and $\tilde{m}$ to identify reference stiffness moduli $G_0^{\textrm{ref}}$ and $E_{50}^{\textrm{ref}}$, have been verified. The first assumption, called here as consistent, is such that that distinct *m* and $\tilde{m}$ values are used, in the second one common *m* exponent (for the range of very small strains) is used for both moduli, in the third one an arbitrary exponent $m=0.5$ is used for both moduli while in the fourth one an averaged exponent $1/2\left( m+ \tilde{m} \right)$ is assumed. Best fits, obtained for gravelly sands and four aforementioned assumptions, concerning power exponents *m*, are shown in Fig. 4. One may notice that all points, for the consistent scheme, are located in a relatively narrow zone along the identified curve. If a unique stiffness exponent *m* is used to identify both $G_0^{\textrm{ref}}$ and $E_{50}^{\textrm{ref}}$ moduli the scatter become slightly bigger with respect to the consistent scheme. Further simplification in which $m=0.5$ is enforced yields a slightly bigger scatter than the first simplified scheme. For an averaged stiffness exponent $1/2(m+\tilde{m})$ the scatter becomes much larger with respect to the first two simplified schemes. Therefore, setting the *m* value in the range of very small strains seems to be optimal from the practical point of view. Another argument for this choice is such that the uncertainty level associated with the *m* value, defined in the range of very small strains, is relatively low (for clean sands very low), while in the range of medium strains it is definitely larger. Another possible solution, at least for gravelly sands, is just to assume $m=0.5$.

**Figure 5:**
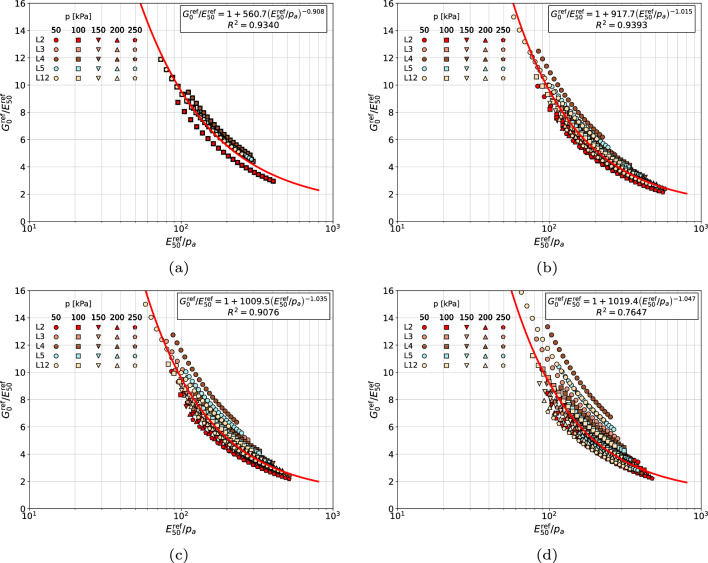
Relations ${G_0^{\textrm{ref}}}/{E_{50}^{\textrm{ref}}}$ vs ${E_{50}^{\textrm{ref}}}/{p_a}$ for coarse and medium sands (in figure (**a**) distinct *m* and $\tilde{m}$ values are used, in figure (**b**) $\tilde{m}=m$ is assumed, in figure (**c**) fixed $m=\tilde{m}=0.5$ is assumed, in figure (**d**) the averaged value $0.5(m+\tilde{m})$ is used).

A similar observations can be made for medium and coarse sands (cf. Fig. 5). In case of the consistent scheme all points are located in a narrow zone along the best fit curve. Once the unique stiffness exponent *m* is used to identify both $G_0^{\textrm{ref}}$ and $E_{50}^{\textrm{ref}}$ moduli the scatter becomes bigger with respect to the consistent scheme. The second simplified scheme, in which $m=0.5$ is assumed for both moduli, yields a significantly larger scatter than the first scheme. The last simplified scheme in which unique averaged stiffness exponent $1/2(m+\tilde{m})$ is used yields the biggest scatter. One may conclude that for the medium and coarse sands the first simplified scheme is optimal.

**Figure 6:**
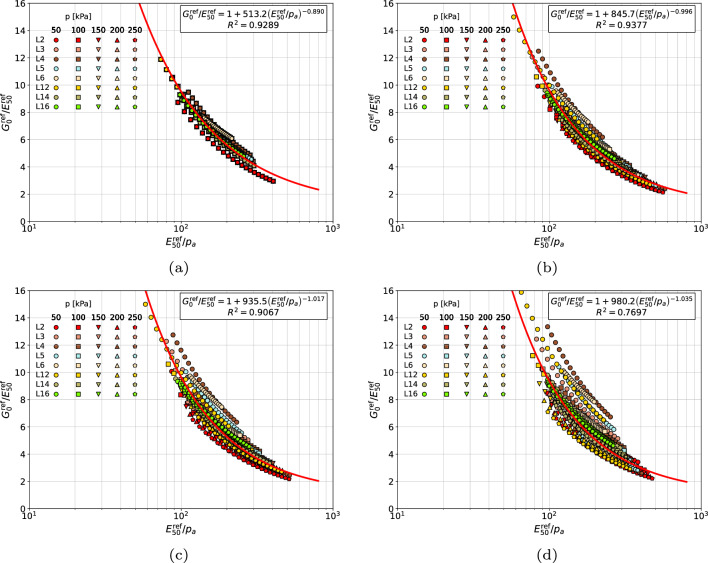
Relations ${G_0^{\textrm{ref}}}/{E_{50}^{\textrm{ref}}}$ vs ${E_{50}^{\textrm{ref}}}/{p_a}$ for gravelly sands, coarse sands and medium sands (in figure (**a**) distinct *m* and $\tilde{m}$ values are used, in figure (**b**) $\tilde{m}=m$ is assumed, in figure (**c**) fixed $m=\tilde{m}=0.5$ is assumed, in figure (**d**) the averaged value $0.5(m+\tilde{m})$ is used).

The resulting correlation formula for gravelly sands, coarse sands and medium sands, collected in one group (cf. Fig. 6), does not differ much with respect to the case of isolated group of coarse and medium sands (cf. Fig. 5). Therefore, from the practical point of view the identified best fit can be used as well.

**Figure 7:**
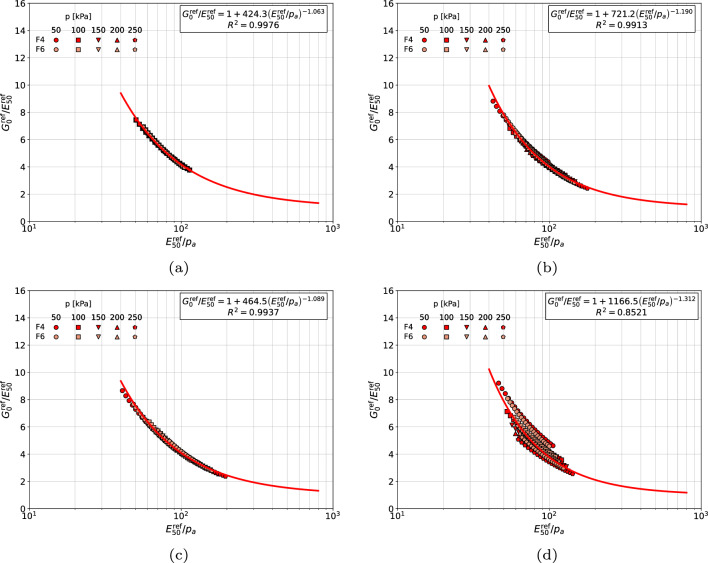
Relations ${G_0^{\textrm{ref}}}/{E_{50}^{\textrm{ref}}}$ vs ${E_{50}^{\textrm{ref}}}/{p_a}$ for fine sands with nonzero fines content (in figure (**a**) distinct *m* and $\tilde{m}$ values are used, in figure (**b**) $\tilde{m}=m$ is assumed, in figure (**c**) fixed $m=\tilde{m}=0.5$ is assumed, in figure (**d**) the averaged value $0.5(m+\tilde{m})$ is used).

In case of fine sands (cf. Fig. 7) the consistent scheme, as well as the two simplified ones, in which the $E_{50}^{\textrm{ref}}$ modulus is reinterpreted using the unique *m*, or $m=0.5$, stiffness exponents yield a very good match between experimental points and derived correlation formulas. The last simplified scheme, in which unique averaged stiffness exponent $1/2(m+\tilde{m})$ is used, yields a significantly larger scatter. It is worth to mention that fine content (FC), in samples F4 and F6, is 11.3% and 19.6% respectively. This is why a significant mismatch between derived formulas is observed among gravelly sands, coarse sands and medium sands, classified in one group, and fine sands.

It is worth to mention that all presented results prove that a strong correlation between $G_0^{\textrm{ref}}/E_{\textrm{50}}^{\textrm{ref}}$ and ${E_{\textrm{50}}^{\textrm{ref}}}/{p_a}$ exists. Moreover, the two simplifications, concerning choice of a unique stiffness exponent *m* value, are acceptable from the practical point of view. However, a certain limit has to be put on the maximum value of $G_0^{\textrm{ref}}/E_{50}^{\textrm{ref}}$. Here $\left( G_0^{\textrm{ref}}/E_{50}^{\textrm{ref}}\right) _{\textrm{max}}\approx 10..12$ is assumed.

**Figure 8:**
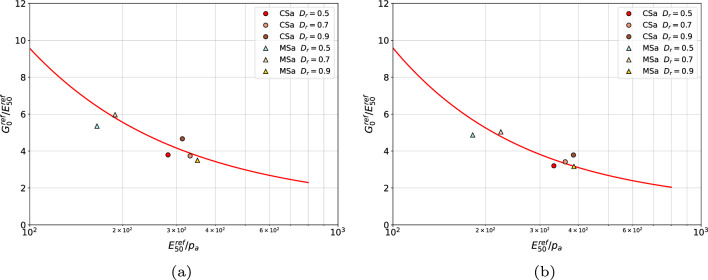
Verification of derived relations ${G_0^{\textrm{ref}}}/{E_{50}^{\textrm{ref}}}({E_{50}^{\textrm{ref}}}/{p_a}$) for coarse and medium Łódź sands (in figure (**a**) distinct *m* and $\tilde{m}$ values are used for the two moduli while in figure (**b**) enforced $\tilde{m}=m$ is used).

Verification of the derived relations has been done based on series of triaxial tests conducted on reconstituted coarse and medium sands from Łódź (Poland). A relatively good match between identified reference moduli ratios $G_0^{\textrm{ref}}/E_{\textrm{50}}^{\textrm{ref}}$ and correlation formula (11) is shown in Fig. 8.

### Relation between reference unloading-reloading and secant stiffness moduli

Another relation that has to be identified using drained triaxial tests results is the one between reference unloading-reloading $E_{\textrm{ur}}^{\textrm{ref}}$ and secant reference $E_{\textrm{50}}^{\textrm{ref}}$ moduli. To analyze this relation triaxial tests must include at least one unloading-reloading cycle induced at a relatively high strength mobilization level $q/q_f$ and conducted till ${q\approx 0}$.

**Figure 9:**
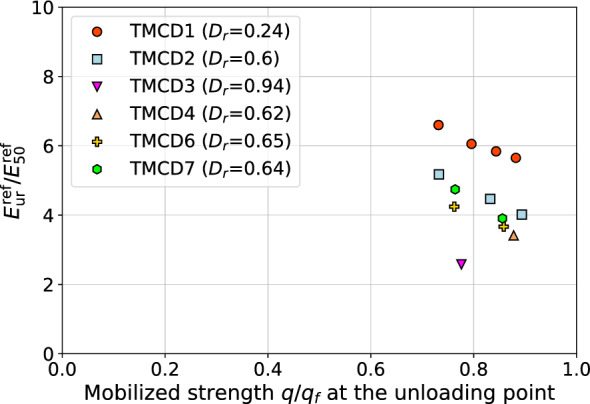
Evolution of the moduli ratio $E_{\textrm{ur}}^{\textrm{ref}}/E_{50}^{\textrm{ref}}$ with the mobilized strength $q/q_f$ level at unloading point.

Determining the unloading-reloading modulus may not be too precise in general because depending on the strength mobilization level, at the unloading point, and total strain $\varepsilon _1$, we do not obtain unique values. This effect is shown in Fig. 9 where six tests for the Karlsruhe sand, published by Wichtmann^8^, are considered. Here unloading-reloading loops in the pre-peak zone $0.7< q/q_f < 1$ are only considered. As it is shown in Fig. 9 the resulting $E_{\textrm{ur}}$ modulus value degrades. In publication^18^ this issue is also reported. Hence we can expect a certain level of uncertainty related to the estimation of this stiffness modulus. In this study the averaged ratios $E_{\textrm{ur}}^{\textrm{ref}}/E_{\textrm{50}}^{\textrm{ref}}$, interpreted in the range $0.7< q/q_f < 1$, are used.

Here the data base includes six tests sands i.e. Karlsruhe sand^8^, Santa Monica sand^19^, Lubiatowo sand^20^, Silica sand^21^, Erksak sand^22^ and Fraser sand^22^. It is worth to mention that results of some of these tests include only one consolidation stress, however, granulometric characteristics $C_u$, $\textrm{FC}$ and relative density $D_r$ are known. Therefore, in all these cases power exponents *m* and $\tilde{m}$ were estimated using formulas (6) and (7).

**Figure 10:**
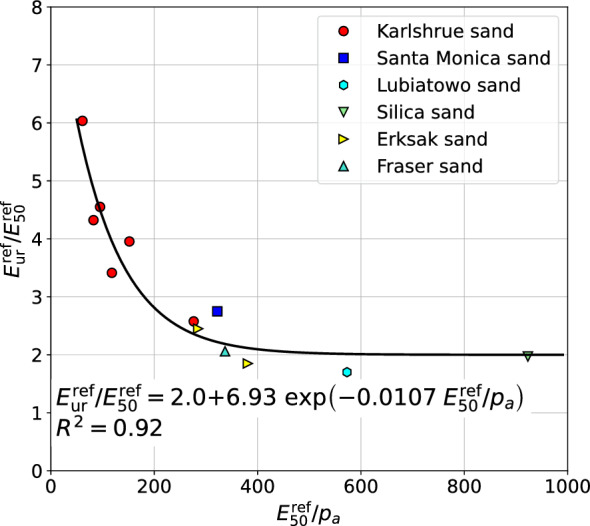
Relation between ${G_0^{\textrm{ref}}}/{E_{50}^{\textrm{ref}}}$ and ${E_{50}^{\textrm{ref}}}/{p_a}$.

Based on this limited set of the data the following correlation formula has been derived (cf. Fig. 10)

12 $$\begin{aligned} E_{\textrm{ur}}^{\textrm{ref}}/E_{50}^{\textrm{ref}}=2+6.93\;\exp {\left( -0.0107 E_{\textrm{50}}^{\textrm{ref}}/p_a \right) } \end{aligned}$$

Taking into account some theoretical aspects of the HS-Brick model (its standard version in fact, without small strain Brick extension), a lower limit $E_{\textrm{ur}}^{\textrm{ref}}/E_{50}^{\textrm{ref}}=2$ has been assumed. A strong correlation between $E_{\textrm{ur}}^{\textrm{ref}}/E_{50}^{\textrm{ref}}$ and $E_{\textrm{50}}^{\textrm{ref}}/p_a$ is well visible. It is worth to mention that notion of the $E_{\textrm{ur}}^{\textrm{ref}}$ modulus is different, in general, for the standard HS model and its enhanced Brick version. This is caused by the fact that the initial unloading/reloading branch in the $q-\varepsilon _1$ relation is strongly influenced by the small strain stiffness. Therefore the $E_{\textrm{ur}}^{\textrm{ref}}$ modulus value for the standard HS model is always higher than for the HS-Brick model, in which strong stiffness variation in the range of small strains is properly represented. This difference is few percents in most cases.

### Relations between reference secant stiffness $E_{50}^{\textrm{ref}}$ and tangent oedometric $E_{\textrm{oed}}^{\textrm{ref}}$ moduli

In the HS-Brick model the $K_0^{\textrm{NC}}$ value and tangent oedometric $E_{\textrm{oed}}$ modulus, set for an arbitrary reference vertical stress $\sigma _{v}^{\textrm{ref}}$, are required to parametrize shape of the cap yield surface and the cap hardening law. It is very important that the pair of values $\{E_{\textrm{oed}}, \sigma _{v}^{\textrm{ref}}\}$ has to be chosen from the virgin consolidation branch of $\varepsilon _v-\sigma _v$ curve, otherwise, the model will not be able to reproduce the assumed $K_0^{\textrm{NC}}$ and the tangent $E_{\textrm{oed}}$ modulus at a given $\sigma _{v}^{\textrm{ref}}$. The Wichtmann’s approximation formula^6^ for the $E_{\textrm{oed}}(e,p)$ modulus, for low and moderate *p* values, yields $E_{\textrm{oed}}$ moduli larger than the corresponding $E_{\textrm{ur}}$ ones. Therefore, to be sure that the $E_{\textrm{oed}}$ modulus is taken from the virgin consolidation line one has to set it for $\sigma _v$ in the range 600...1000 kPa, at least for gravelly sands, coarse sands and medium sands. However, for large vertical stress and low, or very high, $E_{50}^{\textrm{ref}}/p_a$ values the HS-Brick model is not able to reproduce the required $E_{\textrm{oed}}(\sigma _v=8p_a)/E_{50}^{\textrm{ref}}$ moduli ratio. In all these cases the assumed $E_{\textrm{oed}}$ modulus has to be corrected using a dedicated numerical procedure.

Here the group of gravelly sands, medium sands and coarse sands was analyzed in two ways, first as a common group, then gravelly sands were excluded. The general best fit formula for the relation between $E_{\textrm{oed}}^{\textrm{ref}}$ and $E_{\textrm{50}}^{\textrm{ref}}$ moduli can be approximated using the following power law

13 $$\begin{aligned} \dfrac{E_{\textrm{oed}}^{\textrm{ref}}}{E_{\textrm{50}}^{\textrm{ref}}}=A\;\left( E_{\textrm{50}}^{\textrm{ref}} / p_a \right) ^n \end{aligned}$$

where coefficients *A* and *n* have to be optimized for each group of sands, at a given $\sigma _{v}^{\textrm{ref}}$ value. Their values, for gravelly sands, coarse sands and medium sands, grouped together, are given in Table 2, for gravelly sands in Table 3, for medium and coarse sands in Table 4 and for fine sands in Table 5, respectively. Two sets of coefficients *A* and *n*, and coefficient of determination $R^2$, are given in these tables. The first set corresponds to the assumption that modulus $E_{\textrm{50}}^{\textrm{ref}}$ is identified using stiffness exponent $\tilde{m}$, while the second, the exponent *m*.

**Table 2:** Optimized values of coefficients *A* and *n* for gravelly sands, coarse sands and medium sands.

$\sigma _{v}^{\textrm{ref}}/p_a$	*A*	*n*	$R^2$	*A*	*n*	$R^2$
	$\tilde{m}$			*m*		
4	268.2	$-0.756$	0.806	275.8	$-0.774$	0.839
5	272.9	$-0.744$	0.813	309.1	$-0.783$	0.849
6	265.0	$-0.730$	0.830	338.1	$-0.794$	0.859
7	246.6	$-0.713$	0.852	363.9	$-0.806$	0.865
8	219.6	$-0.691$	0.870	387.5	$-0.820$	0.868

**Table 3:** Optimized values of coefficients *A* and *n* for gravelly sands.

$\sigma _{v}^{\textrm{ref}}/p_a$	*A*	*n*	$R^2$	*A*	*n*	$R^2$
	$\tilde{m}$			*m*		
4	356.3	$-0.799$	0.996	383.2	$-0.816$	0.992
5	362.9	$-0.784$	0.996	401.1	$-0.808$	0.991
6	355.7	$-0.770$	0.996	406.0	$-0.800$	0.988
7	338.2	$-0.754$	0.995	401.2	$-0.793$	0.983
8	313.2	$-0.738$	0.992	388.9	$-0.787$	0.976

**Table 4:** Optimized values of coefficients *A* and *n* for medium and coarse sands.

$\sigma _{v}^{\textrm{ref}}/p_a$	*A*	*n*	$R^2$	*A*	*n*	$R^2$
	$\tilde{m}$			*m*		
4	241.0	$-0.740$	0.757	168.6	$-0.691$	0.763
5	244.2	$-0.728$	0.773	163.1	$-0.677$	0.800
6	235.9	$-0.713$	0.806	159.0	$-0.669$	0.856
7	218.0	$-0.695$	0.851	155.8	$-0.667$	0.917
8	192.3	$-0.672$	0.902	152.5	$-0.668$	0.963

**Table 5:** Optimized values of coefficients *A* and *n* for fine sands with nonzero fines content.

$\sigma _{v}^{\textrm{ref}}/p_a$	*A*	*n*	$R^2$	*A*	*n*	$R^2$
	$\tilde{m}$			*m*		
4	45.7	$-0.445$	0.990	39.6	$-0.446$	0.944
5	53.3	$-0.445$	0.990	46.8	$-0.457$	0.918
6	60.5	$-0.445$	0.991	54.3	$-0.469$	0.896
7	67.3	$-0.445$	0.991	61.9	$-0.479$	0.879
8	73.8	$-0.445$	0.991	69.7	$-0.489$	0.866

**Figure 11:**
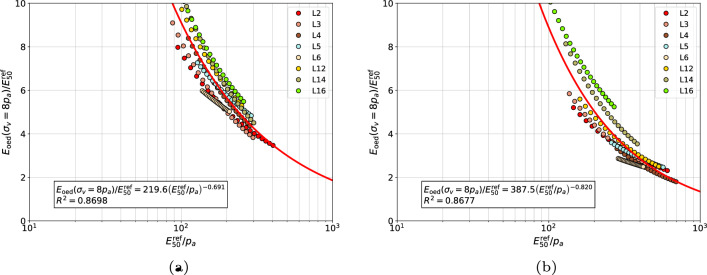
Relation between ${E_{\textrm{oed}}^{\textrm{ref}}}\left( \sigma _v=8\;p_a \right) /{E_{50}^{\textrm{ref}}}$ and ${E_{50}^{\textrm{ref}}}/{p_a}$ for gravelly sands, coarse sands and medium sands (in figure (**a**) the $E_{\textrm{50}}^{\textrm{ref}}$ modulus was computed using exponent $\tilde{m}$ while in figure (**b**) the exponent *m*, respectively).

**Figure 12:**
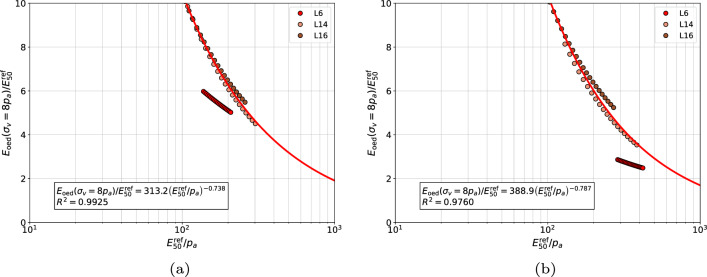
Relation between ${E_{\textrm{oed}}^{\textrm{ref}}}\left( \sigma _v=8\;p_a \right) /{E_{50}^{\textrm{ref}}}$ and ${E_{50}^{\textrm{ref}}}/{p_a}$ for gravelly sands (in figure (**a**) the $E_{\textrm{50}}^{\textrm{ref}}$ modulus was computed using exponent $\tilde{m}$ while in figure (**b**) the exponent *m*, respectively).

**Figure 13:**
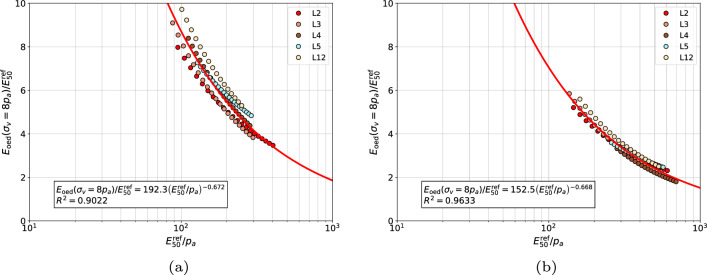
Relation between ${E_{\textrm{oed}}^{\textrm{ref}}}\left( \sigma _v=8\;p_a \right) /{E_{50}^{\textrm{ref}}}$ and ${E_{50}^{\textrm{ref}}}/{p_a}$ for medium and coarse sands (in figure (**a**) the $E_{\textrm{50}}^{\textrm{ref}}$ modulus was computed using exponent $\tilde{m}$ while in figure (**b**) the exponent *m*, respectively).

**Figure 14:**
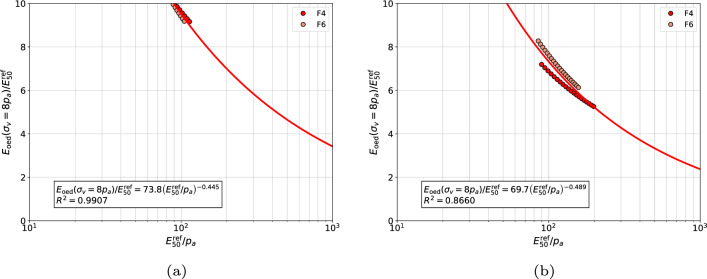
Relation between ${E_{\textrm{oed}}^{\textrm{ref}}}\left( \sigma _v=8\;p_a \right) /{E_{50}^{\textrm{ref}}}$ and ${E_{50}^{\textrm{ref}}}/{p_a}$ for fine sands with nonzero fines content (in figure (**a**) the $E_{\textrm{50}}^{\textrm{ref}}$ modulus was computed using exponent $\tilde{m}$ while in figure (**b**) the exponent *m*, respectively).

The proposed correlations for each group of sands at $\sigma _{v}^{\textrm{ref}}/p_a=8$ are visualized in Figs. 11, 12, 13 and 14, respectively. In general, no mater whether *m* or $\tilde{m}$ stiffness exponent is used, the proposed power law relatively well approximates the measurements.

## Estimating elastic stiffness in the range of very small strains using CPTU tests

The CPTU test is frequently used to estimate elastic moduli *E* and $G_0$, used later on in serviceability limit states assessment. The $E_0$ modulus appears as an explicit parameter in the HS-Brick model while the *E* modulus is in most cases somewhere between the $E_{\textrm{ur}}$ and $E_{\textrm{50}}$. Therefore it is highly recommended to perform seismic SCPTU test to calibrate correlations for shear wave velocity adjusted to local conditions. It has to be emphasized that the $G_{0}$ modulus is common for the SCPTU and triaxial tests, enhanced by the shear wave velocity measurement. In practical applications the SCPTU is not as frequently done as the standard CPTU test. Hence, searching for accurate CPTU correlations enabling shear wave velocity $V_s$ estimation is of great importance. A comprehensive study of the existing CPTU correlations for $V_s$, for sands, is given in^7^. In this publication the six most popular correlations ie. the one by Hegazy and Mayne^23^, Mayne^24^, Andrus et al.^25^, Robertson^26^, McGann et al.^27^ and Ahmed^28^ are analyzed. Some of these correlations are based on dimensionless parameters such as normalized tip resistance $Q_{tn}$ and soil behavior type material index $I_c$ while others on dimensional quantities like effective vertical stress $\sigma _{v}^{\prime }$, total vertical stress $\sigma _{v}$, sleeve friction $f_s$ and corrected tip resistance $q_{t}$. In the aforementioned publication the author verified performance of these correlations and indicated that the prediction error of the resulting stiffness modulus $G_0$ is ranging from $-30$ to 335%. The inconsistent stress normalization used in the behavioral index $I_c$ and ratio $G_0/\sigma _{v}^{\prime }$ was indicated by the author as a major source of such high discrepancies. For uniform sand layers the standard $I_c$ index is practically constant while the ratio $G_0/\sigma _{v}^{\prime }$ depends on the $\sigma _{v}^{\prime }$. Whole study conducted by Ahmed^7^ was based on the data base, consisting of 15 high quality undisturbed sand samples from Japan, Canada, Norway, China and Italy^29^. The reasoning was that ${G_0}/{p_a}$ is proportional to $p^{\prime }/{p_a}$

14 $$\begin{aligned} \dfrac{G_0}{p_a} \propto \left( \dfrac{p^{\prime }}{p_a} \right) ^m \end{aligned}$$

where the mean effective stress is denoted by $p^{\prime }$ and an arbitrary reference stress $p_a$ is equal to the atmospheric pressure value.

Then, as the mean effective stress $p^{\prime }$ is proportional to the $\sigma _{v}^{\prime }$ through the relation

15 $$\begin{aligned} p^{\prime } =\dfrac{1+2\;K_0}{3} \sigma _{v}^{\prime } \end{aligned}$$

the following proportionality has to be hold

16 $$\begin{aligned} \dfrac{G_0}{p_a} \propto \left( \dfrac{\sigma _{v}^{\prime }}{p_a} \right) ^m \end{aligned}$$

Assuming that the stiffness exponent *m* is expressed as a linear function of the $I_c$ index and fraction $\sigma _{v}^{\prime }/p_a$, and taking into account Wichtmann’s formula (5) the following correlations were derived by Ahmed^7^

17 $$\begin{aligned} m= & {} 0.167\;I_c-0.002{\sigma _{v}^{\prime }}/{p_a}+0.232 \end{aligned}$$

18 $$\begin{aligned} {G_0}= & {} 5300\;{\sigma _{v}^{\prime }}\;\exp {(-1.25\;I_{cc})}\;f(F_r) \end{aligned}$$

where

19 $$\begin{aligned} f(F_r)= & {} 0.21\;F_r+0.85 \ge 1.0 \end{aligned}$$

20 $$\begin{aligned} I_{cc}= & {} \sqrt{\left( 3.47-\log {Q_{tc}}\right) ^2+\left( 1.22+\log {F_r} \right) ^2} \end{aligned}$$

21 $$\begin{aligned} Q_{tc}= & {} \dfrac{Q_{tn}}{\left( \sigma _{v}^{\prime }/p_a \right) ^{1-m}} \end{aligned}$$

In the above expressions normalized friction ratio (in percent) is denoted as $F_r$, while the corrected normalized tip resistance and corrected soil behavior type material index are denoted as $Q_{tc}$ and $I_{cc}$, respectively.

In this study a different correlation formula for the stiffness exponent is used (Eq. 6), therefore, the resulting correlation between *m*, $I_c$ and ${\sigma _{v}^{\prime }}$ is expressed as follows

22 $$\begin{aligned} m=0.110\;I_c+0.0006\;{\sigma _{v}^{\prime }}/{p_a}+0.323 \end{aligned}$$

It is well visible that the stress dependent term in Eq. (22) is negligible and can be skipped in the optimization procedure. This modification yields a much simpler formula for the stiffness exponent

23 $$\begin{aligned} m=0.111\;I_c+0.322 \end{aligned}$$

The following correlation formula for the $G_0$ modulus was derived using particle swarm optimization

24 $$\begin{aligned} {G_0}=6535\;{\sigma _{v}^{\prime }}\;\exp {(-1.470\;I_{cc})}\;f(F_r) \end{aligned}$$

where

25 $$\begin{aligned} f(F_r)=0.421\;F_r+0.897 \end{aligned}$$

**Figure 15:**
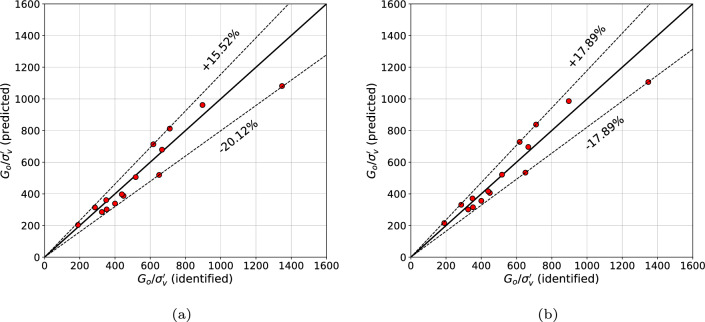
Predicted vs measured ${G_0}/{\sigma _{v}^{\prime }}$ values for 15 sands from data base by Mayne^29^ (in figure (**a**) result for unconstrained optimization is shown while in figure (**b**) for the constrained one).

The resulting prediction error for the $G_0$ modulus is ranging from $-20.1$ to 15.5% (cf. Fig. 15a).

Another formula (26), for the $G_0$ modulus, is obtained, by enforcing equal error margins. In this case the smallest prediction error is within the range $-17.9$ to 17.9% (cf. Fig. 15b).

26 $$\begin{aligned} {G_0}=7127\;{\sigma _{v}^{\prime }}\;\exp {(-1.432\;I_{cc})}\;f(F_r) \end{aligned}$$

where

27 $$\begin{aligned} f(F_r)=0.343\;F_r+0.812 \end{aligned}$$

Both formulas (26) and (24) have to be used with caution at very shallow depths where mean effective stresses are low. Moreover, as it has been underlined in Ref.^7^, the above formulas do not apply to the aged and/or cemented sands.

**Figure 16:**
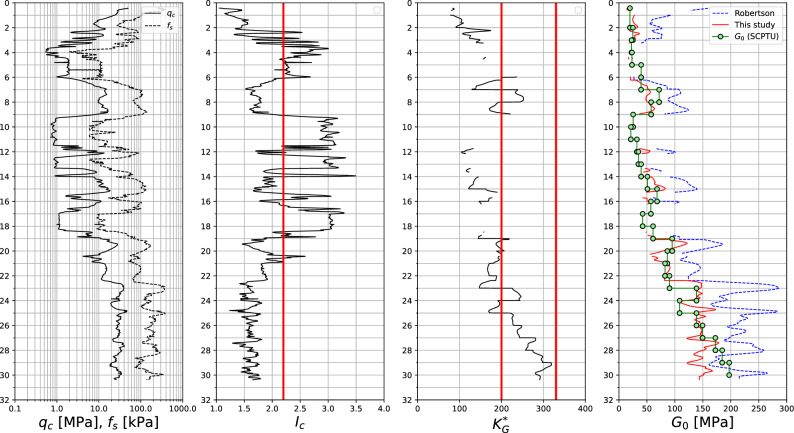
SCPTU-1 test: $q_c$, $f_s$, $I_c$, $K_G^{*}$ and predicted vs measured $G_0$ moduli profiles.

**Figure 17:**
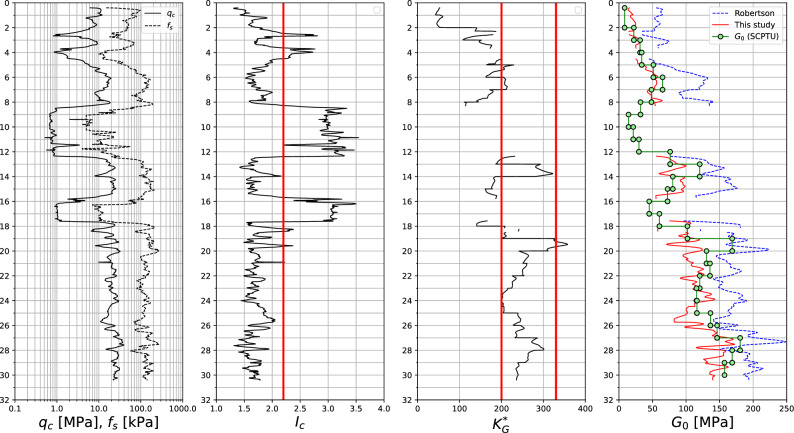
SCPTU-2 test: $q_c$, $f_s$, $I_c$, $K_G^{*}$ and predicted vs measured $G_0$ moduli profiles.

**Figure 18:**
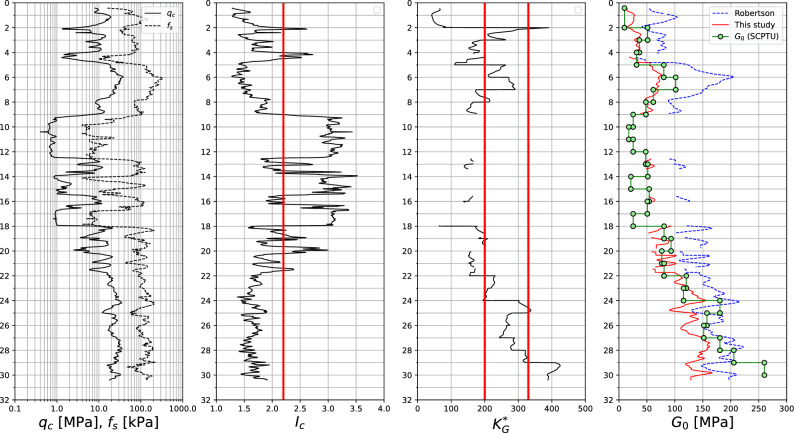
SCPTU-3 test: $q_c$, $f_s$, $I_c$, $K_G^{*}$ and predicted vs measured $G_0$ moduli profiles.

The formula (26) is verified here using results of the 3 SCPTU tests conducted in sandy subsoil in Gdańsk (Poland). The considered subsoil, till depth 19 m, consists of fine/medium sand and mud/clayey sand layers, then fine/medium/gravelly sands occur. The maximum sounding depth is about 30 m. Interpretation of these tests is shown in Figs. 16, 17, and 18 where quantities $q_c$, $f_s$, $I_c$ and $K_G^{*}$, as well as the measured vs interpreted $G_0$ moduli, using formula (26), and the one by Ref.^30^, are visualized. The $K_G^{*}$ parameter, expressing significance of the microstructure (bonding/cementation and aging), is computed as follows^30^

28 $$\begin{aligned} K_G^{*}=\left( \dfrac{G_0}{q_t-\sigma _{v}} \right) Q_{tn}^{0.75} \end{aligned}$$.

One may notice that for the low value of the $K_G^{*}$ parameter the formula by Robertson may yield a significant overshoot for the $G_0$ modulus, while the formula (26) yields $G_0$ values closer to the measured ones. When the $K_G^{*}$ tends to the value 330 or more, the formula (26) practically always underestimates the $G_0$ modulus. This effect is most likely caused by the effect of the aging.

## Stiffness degradation in the range of small strains

In Ref.^31,32^ the following formula for the degradation curve $G/G_0(\gamma )$ is proposed (FC is expressed here in decimal notation)

29 $$\begin{aligned} \dfrac{G}{G_{0}}=\dfrac{1}{1+a\dfrac{\gamma }{\gamma _r}} \end{aligned}$$

where

30 $$\begin{aligned} a= & {} \left( 1+0.847\ln {(C_u)}\right) \exp {\left( 2.05 \;\textrm{FC} \right) } \end{aligned}$$

31 $$\begin{aligned} \gamma _r= & {} \dfrac{\tau _{\textrm{max}}}{G_0} \end{aligned}$$

The maximum shear stress at in situ conditions can be expressed as follows

32 $$\begin{aligned} \tau _{\textrm{max}}=\max \left( \dfrac{\sigma _1-\sigma _3}{2}\right) =\dfrac{\sigma _v^{\prime }(1-K_a)}{2} \end{aligned}$$

where

33 $$\begin{aligned} K_a=\dfrac{1-\sin \phi ^{\prime }}{1+\sin \phi ^{\prime }} \end{aligned}$$

The $C_u$ and FC are easily obtained from the granulometric curve, while the $G_0$ modulus, at a given depth, has to be interpreted from the measured shear wave velocity, or it can be estimated using formula (24), or (26). The $C_u$ and FC values can be estimated as well using the inverse relation between power exponent *m* and $I_c$ index, once the fine content FC is known. On the other hand the FC value can be estimated using CPTU correlations proposed by Robertson and Wride^33^, Boulanger and Idriss^34^, Agaiby and Mayne^35^ or by Yi^36^. From the practical point of view an averaged value can be used unless the deviation from the mean value of one of the aforementioned correlations is too high.

In the HS-Brick model the adopted stiffness degradation curve is defined as follows

34 $$\begin{aligned} \dfrac{G}{G_{0}}=\dfrac{1}{1+\tilde{a}\dfrac{\gamma }{\gamma _{0.7}}} \end{aligned}$$

where $\tilde{a}=0.385$ is assumed.

By comparing the two expressions (29) and (34) the following formula for the HS-Brick model parameter $\gamma _{0.7}$ is derived (FC in decimal notation)

35 $$\begin{aligned} \gamma _{0.7}=\dfrac{\tilde{a}}{a}\gamma _r=\dfrac{0.385}{\left( 1+0.847\ln {(C_u)}\right) \exp {\left( 2.05 \;\textrm{FC} \right) } }\;\dfrac{\sigma _v^{\prime }(1-K_a)}{2G_{0}} \end{aligned}$$

It has to be emphasized that in the HS-Brick model the $\gamma _{0.7}$ parameter is kept constant. Therefore, it seems to be optimal to adjust its value referring to the in situ stress state. In that case it will vary with depth. It is also worth to mention that the $\gamma _{0.7}$ parameter can be estimated using exclusively the CPT test results by means of the inverse relations derived from equations (6), (23) and the estimated FC value.

## Estimating peak friction and dilatancy angles from CPTU tests

To complete set of HS-Brick model parameters peak friction and dilatancy angles have to be established. These parameters can easily be estimated based on the CPTU test results using one of the existing correlations (here Robertson formula is used)^30^

36 $$\begin{aligned} \phi ^{\prime }_{\textrm{peak}}= & {} \phi ^{\prime }_{\textrm{cv}}+15.84\;\log {}Q_{\textrm{tn,cs}}-26.88 \end{aligned}$$

37 $$\begin{aligned} \psi= & {} \phi ^{\prime }_{\textrm{peak}}-\phi ^{\prime }_{\textrm{cv}} \end{aligned}$$

where $\phi ^{\prime }_{\textrm{cv}}\approx 33^o$ for quartz sands^16^.

## Conclusions

In the article a practical method of estimating Hardening Soil-Brick parameters, using CPTU test results and experimental evidence, derived from drained triaxial and oedometric laboratory tests, has been proposed and verified.

Based on the rich set of the high quality triaxial and oedometric tests conducted on several classes of sands by Wichtmann et al.^6^ it has been shown that a strong correlation between the reference small strain stiffness modulus $G_0^{\textrm{ref}}$ and the secant reference stiffness modulus $E_{50}^{\textrm{ref}}$ exists and can easily be approximated, using the power law, with a high coefficient of determination. The major difficulty in the HS-Brick model, in which the power stiffness exponent *m* has to be assumed as a constant, has been overcome by remapping reference secant stiffness moduli $E_{50}^{\textrm{ref}}$ using the *m* value corresponding to the small strain regime. In addition a unique relation between the ratio $E_{\textrm{ur}}^{\textrm{ref}}/E_{50}^{\textrm{ref}}$ and dimensionless stiffness parameter $E_{50}^{\textrm{ref}}/p_a$, using set of triaxial tests conducted on different sands, has been established with a very high coefficient of determination. It has also been shown that there exists a strong correlation between ratio $E_{\textrm{oed}}^{\textrm{ref}}/E_{50}^{\textrm{ref}}$ and $E_{50}^{\textrm{ref}}/p_a$ at different reference vertical effective stresses. Following the original work by Ahmed and his idea of consistent stress normalization when estimating small strain stiffness modulus $G_0$, using the CPTU tests results, a simple correlation formula for the stiffness exponent *m* has been derived in which effect of the vertical effective stress can be neglected. Based on the data set published by Mayne and the identified formula for the *m* parameter, a more accurate formula for the $G_0$ modulus has been derived, using particle swarm optimization method. For the best solution found the estimation error is bound in the range $-17.9 \cdots +17.9$% versus 20% when using Ahmed’s formula. To make the estimation of the HS-Brick model parameters complete, a relatively simple method for estimating the $\gamma _{0.7}$ parameter, based on the CPTU tests, has been proposed. All the results published in this article may help to conduct advanced numerical analyses of real life soil-structure interaction problems for non-aged/uncemented coarse grained soils and can be very helpful in further studies concerning random fields and reliability based design procedures.

## Data Availability

Some datasets used and/or analysed during the current study may be available from the author on reasonable request. The exact location of places from which samples were gathered to conduct triaxial tests results on Łódź sands, as well as the exact place of the SCPTU tests in Gdańsk, are confidential.
